# Prevalence of Cannabis Use and Factors Related to Hospitalizations in the United States: A Population-Based Study Using National Inpatient Sample Between 2012 and 2018

**DOI:** 10.7759/cureus.28361

**Published:** 2022-08-24

**Authors:** Saanie Sulley, Memory Ndanga, Abimbola K Saka

**Affiliations:** 1 Health and Biomedical Informatics, Independent Researcher, Washington DC, USA; 2 Health Information Management, Rutgers University, Newark, USA; 3 Translational Research, Independent Researcher, Toronto, CAN

**Keywords:** cannabis legalization, cannabis abuse, cannabis use disorder, marijuana use and hospitalization, cannabis use

## Abstract

Introduction: Cannabis use has been associated with adverse outcomes among adults and adolescents. As more states legalize or consider legalization, it is imperative to understand cannabis-related hospitalizations among the US population. This study is aimed at understanding the prevalence of cannabis-related hospitalizations using a nationally representative sample.

Methods: Using the National Inpatient Sample (NIS) available through the Healthcare Cost and Utilization Project (HCUP), we included all hospitalizations that met the inclusion criteria of documented history of cannabis use and those with any cannabis diagnosis as the reason for hospitalization between 2012 and 2014, and 2016 and 2018 using listwise deletion methods. Cannabis use was identified based on International Classification of Disease (ICD 9 & 10) codes (304.3X, 305.2X) (F12.XXX) for 2012-2014 and 2016-2018, respectively. We included both primary and secondary diagnoses among hospitalized patients. We further analyzed the relationship between cannabis-related diagnoses, race and ethnicity cases, household income, region, age group, rural-urban demographics, and sex.

Results: A weighted total of 2,099,665 and 1,023,325 patients with a history of cannabis use were identified for the period of 2012-2014 and 2016-2018, respectively. The primary reason for presentation among a majority of patients was related to mental health, alcohol, HIV, trauma, burns, and toxic effects of drugs for all included years. The rate of the presentation was highest among individuals 12-24-years-old (351, 846) and 25-34-years-old (255 and 563) per 10,000 presentations between 2012-2014 and 2016-2018, respectively. The highest rate of increase by race and ethnicity was observed among Native Americans (227 and 457), Black (287 and 468), and others (125 and 214) during 2012-2014 and 2016-2018, respectively. The highest observations were in the East North Central, West North Central, Mountain, and Pacific Regions of the United States. The highest presentation rates were observed among males with no insurance coverage and populations in the lowest income quartiles.

Conclusion: Cannabis-related hospitalization increased significantly over the years, and presentations are not isolated to areas with cannabis legalization. The high presentation rate among individuals with mental and alcohol necessitates the development of strategies to educate and mitigate potential causes of hospitalization among all age groups and races or ethnicity.

## Introduction

The legalization of cannabis use has found itself at the center stage of many debates in American politics. For the past few years, many states have been setting the stage for liberalization policies and allowing for broader use of medical marijuana, classified as a schedule 1 drug, since the 1970s. It was illegal to possess, use, buy, sell, or cultivate cannabis in all United States. The establishment of the 2018 United States farm bill, under federal law, enabled several states to re-examine policies since the bill removed hemp seeds from the statutory definition of marijuana.

A decade ago, marijuana was illegal across the United States, but the plant is now legal for medicinal purposes in 36 states and completely legal in 15 [1]. Even with the growing acceptance of marijuana use, cannabis use is associated with many adverse events [2-5]. Research has shown that recreational marijuana use is associated with a 17% increased likelihood of acute ischemic stroke hospitalization [6]. There has also been noticeable research on the association between cannabis use and harmful effects like cardiovascular, cerebrovascular, and neurological complications in various age groups [7-10]. More than half of the patients presented psychiatric disorders, and almost a third presented respiratory system disorders or cardiovascular disorders [7]. Chronic cannabis use has also been attributed to worsening hospitalization outcomes in arrhythmic patients. More clinical studies are needed to study the causal association between these conditions due to the rising mortality risk [11]. Retrospective studies have indicated that cannabis use may accelerate the onset of schizophrenia [12,13].

Some of the hospitalizations have been tied to the use of marijuana edibles. Users are likely to digest more because it takes longer to feel the effects of the drug and will likely result in seeking medical attention due to acute intoxication [14]. A report by the Colorado Department of Public Health and Environment shows that there has been an increase in marijuana-related hospital codes [15]. It is estimated that the portion of hospitalizations with a marijuana code increased among 9-17-year-olds but tripled among 18-25-year-olds, reaching 8.1% in 2014-2015 [16].

Though medical marijuana has seen progress in patient outcomes, medical marijuana users have been associated with more medical problems. A significant proportion reported >15 days of medical problems in the past month [17]. Research has also shown that Americans are using marijuana to treat medical conditions despite a lack of evidence of efficacy [18]. The literature review does show that there is a need for further research to assess the potential adverse and beneficial effects of marijuana use on health outcomes [19-21]

Since there have been significant changes in the way several states view recreational and medical marijuana. This research aims to analyze hospitalization trends for cannabis use in terms of demographic characteristics and geographical region, comorbidity, length of stay (LOS), and total charges during hospitalization. The research will aid in understanding marijuana policies' effects on the healthcare system. It will also allow researchers to access the need for further studies and medical education regarding medicinal marijuana.

## Materials and methods

We utilized the National Inpatient Sample (NIS) dataset sponsored by the Agency for Healthcare Research and Quality (AHRQ) under the Healthcare Cost and Utilization Project (HCUP). We included all hospitalizations between 2012-2018 and excluded missing cases for variables of interest. The weighted NIS data contains more than 35 million hospitalizations annually [22]. We identified cannabis use based on and International Classification of Disease (ICD 9 & 10) codes (304.3X, 305.2X) (F12.XXX), respectively. We included primary, principal, and secondary diagnoses that met the inclusion criteria. Principal or primary cannabis-related diagnoses were mainly responsible for admissions, while secondary diagnoses were not the main cause of hospitalization among the patient population. We analyzed the presentations of cannabis use disorder based on their corresponding ICD 9/10 codes and the major categories associated with patient presentations using the Major Diagnostic Category (MDC). This descriptive study compared the prevalence of cannabis diagnosis and race and ethnicity, household income, region, age group, rural-urban demographics, and sex.

We utilized the data that met the primary and secondary diagnoses of cannabis use to generate descriptive statistics to understand the prevalence among the selected variables. We conducted further analysis to evaluate the presence of cannabis and severity at a presentation by region. We utilized IBM Statistical Package for Social Sciences (SPSS) version 23.0 (IBM Corp., Armonk, NY) for analyses related to this study. All analyses of this data set adhered to recommended HCUP guidelines. We analyzed ICD-9 (2012-2014) and ICD-10 (2016-2018) separately. We excluded data from 2015 because of ICD9/ICD10 switch in the United States in October 2015. Weighted presentation rates were calculated and graphed by patient and demographic characteristics using Power BI 2019 (Microsoft Corporation, Redmond, WA). We examined the differences in hospitalizations with any indication of cannabis use based on the ICD 9/10 codes. We further calculated and analyzed the differences in the rate of cannabis use based on the ICD 9 and 10 inclusion criteria and relationship with the sociodemographic variables of interest, including sex, age, region, race or ethnicity, emergency room (ER) use, median household income, and the primary reason for hospitalizations for individuals with any indication of cannabis use. A chi-squared test was used to assess the relationship between the cannabis use indicator and variables of interest. Missing cases among selected variables were excluded in this study.

## Results

**Figure 1:**
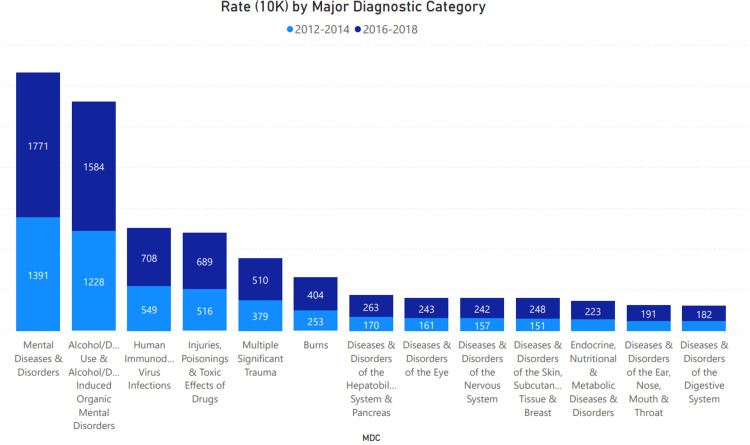
Most Common Primary Reason for Hospitalization by Major Diagnostic Category (MDC) categorized by 2012-2014 (ICD-9) and 2016-2018 (ICD-10). As shown here the primary reason for hospitalization was related to mental disorders, alcohol and drug-related diagnoses, HIV, poisoning, significant trauma, and burns.

The primary reason for presentation among individuals with cannabis-related diagnosis (MDC) among the population was associated with mental health and alcohol-related diagnoses. The presentation rate increased between 2012-2018, as shown in Figure 1. The rate among HIV-infected individuals with multiple significant trauma and burns increased during the same period. The fourth highest primary reason for the presentation was related to the toxic effects of drugs at a rate of 516 (2012-2014) and 269 (2016-2018), respectively. 

**Figure 2:**
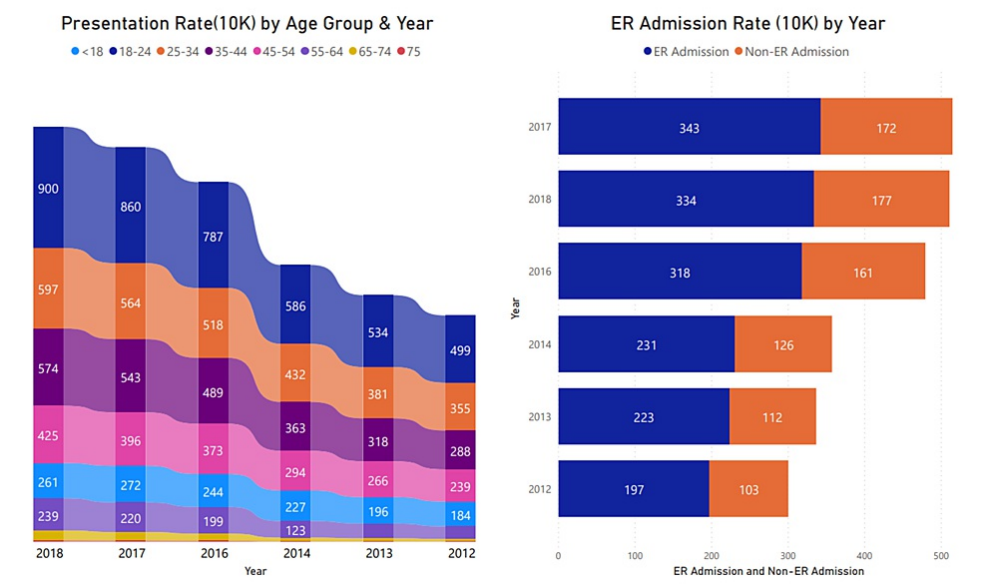
Cannabis Presentation Rate by Age Group and ER as the source of Admission As shown here, there was a consistent increase in the presentation rate across all age groups, especially among 18-24 and 25-34 years. The rate of admissions for patients with cannabis use was higher across all years from 197 in 2012 to 334 in 2018. ER: Emergency room

The highest presentation rates are among individuals ages 18-24 years and 25-34 years across all years included in this study, as shown in Figure 2. The older population (55+ years old) that met the inclusion criteria had a higher rate of chronic conditions in the Diseases & Disorders of the Musculoskeletal System & Connective Tissue. Trauma and burns were more frequent among the younger population (<18-34 years old) using cannabis compared to other age groups. Emergency services utilization among this group also increased between 146 and 322 per 10,000 presentations. 

**Figure 3:**
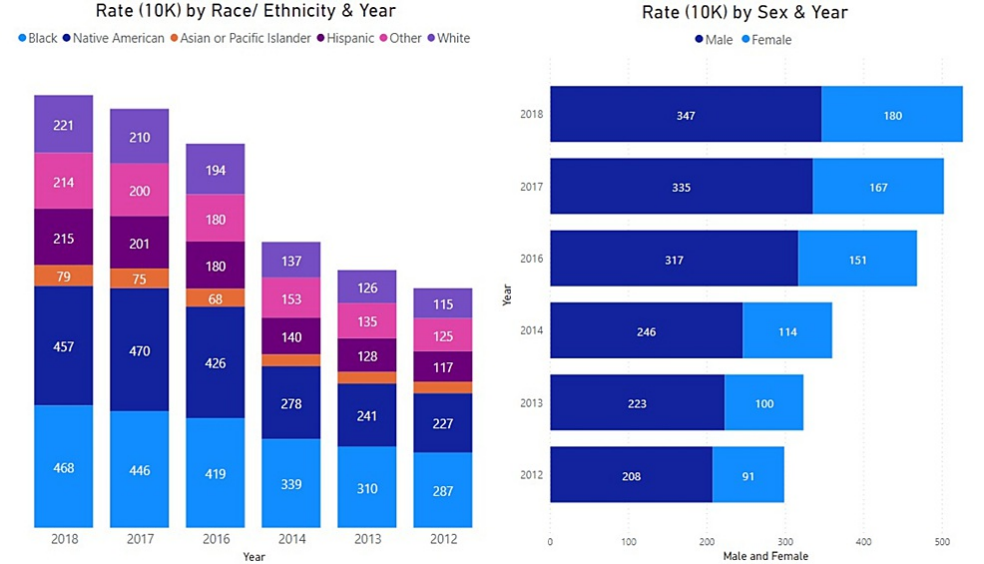
Cannabis Presentation Rate by Race/Ethnicity and Sex As shown here, there was a consistent increase in presentation rates across all races and ethnicities with the highest observed rates among Black/African American and Native American populations. Males were more likely to present with cannabis-related diagnoses compared to females at a rate of 208 in 2012 to 347 in 2018 per 10,000 presentations.

The presentation rate increased in both males and females, as shown in Figure 3. The presentation rates have increased across all racial groups, with the highest rate of presentation among Native Americans (227 and 457), Black (287 and 468), and others (125 and 214) duirng the 2012-2014 and 2016-2018 ranges, respectively.

As shown in Table 1, cannabis users by severity at presentation showed an increased prevalence and rate among individuals with major and extreme loss of function during 2012-2014 and 2016-2018, respectively. 

**Table 1:** Descriptive Statistics of cannabis-related hospitalizations rate (per 10,000) in the United States during 2012-2014 and 2016-2018 MDC:  Major diagnostic category; ER: Emergency room

Variables of Interest	Cannabis Related Disorders		P-value (Sig)
	2012-2014	2016-2018	
Inclusion (N-weighted)	2099665	1223200	
Most Common MDC	N (Rate per 10K)	N (Rate per 10K)	
Mental Diseases & Disorders	745604(1391)	780415(1771)	
Alcohol/Drug Use & Alcohol/Drug Induced Organic Mental Disorders	194720(1228)	220130(1584)	
Human Immunodeficiency Virus Infections	10930(549)	10145(708)	
Injuries, Poisonings & Toxic Effects of Drugs	104770(516)	111000(689)	
Multiple Significant Trauma	11915(379)	15150(510)	
Burns	3045(253)	3835(404)	
Diseases & Disorders of the Hepatobiliary System & Pancreas	66835(170)	82525(263)	
Age Group			<0.001
<18	60515(174)	89930(256)	
18-24	98395(351)	434590(846)	
25-34	341810(255)	636510(563)	
35-44	486615(218)	441075(537)	
45-54	301880(189)	403695(398)	
55-64	351425(94)	331830(219)	
65-74	229335(44)	105955(64)	
75+	113595(32)	16600(8)	
Sex			
Female	895095(89)	963525(166)	
Male	1204235(162)	1496445(335)	<0.001
Race/Ethnicity			
White	1143985(105)	1385700(227)	
Black	541575(216)	693330(497)	
Hispanic	180590(94)	255630(241)	
Asian or Pacific Islander	19225(43)	23665(93)	<0.001
Native American	17330(162)	30305(516)	
Other	64390(113)	71555(252)	
ER Utilization			
ER Code Present	1258610(146)	1653225(322)	
ER Code Not Present	841055(96)	807155(171)	<0.001
Hospital Region			
New England	115285(139)	114315(239)	
Middle Atlantic	358685(141)	347255(239)	
East North Central	434245(156)	424915(270)	
West North Central	175455(142)	169235(274)	<0.001
South Atlantic	448935(123)	504640(235)	
East South Central	130990(106)	144385(199)	
West South Central	186270(87)	201765(162)	
Mountain	93375(85)	164615(278)	
Pacific	156425(80)	389255(271)	
Admission Day			
Admitted Monday-Friday	1698294(148)	1896475(233)	
Admitted Saturday-Sunday	495680(170)	563895(267)	
Primary Payer (%)			
Medicare	472000(68)	430980(102)	
Medicaid	714940(188)	1116035(475)	
Private Insurance	499490(94)	521430(175)	
Self-Pay	272390(345)	265490(650)	<0.001
No charge	30435(418)	24235(751)	
Other	105355(193)	97240(336)	
Rural Urban Code			
"Central" counties of metro areas of >=1 million population	510215(127)	848100(273)	
"Fringe" counties of metro areas of >=1 million population	356840(107)	496230(200)	
Counties in metro areas of 250,000-999,999 population	319990(115)	522690(250)	
Counties in metro areas of 50,000-249,999 population	143750(112)	231585(249)	<0.056
Micropolitan counties	146370(109)	197000(215)	
Not metropolitan or micropolitan counties	93140(94)	114710(169)	
Median Household Income			<0.001
0-25th percentile	799245(152)	971665(316)	
26th to 50th percentile (median)	516230(118)	617005(234)	
51st to 75th percentile	410750(101)	477080(200)	
76th to 100th percentile	300300(87)	313095(157)	
Severity Risk			<0.001
No class specified	1085(68)	1055(166)	
Minor loss of function (includes cases with no comorbidity or complications)	635535(122)	603345(197)	
Moderate loss of function	981845(169)	1213990(328)	
Major loss of function	386085(106)	500020(192)	
Extreme loss of function	95115(95)	141970(157)	
Mortality Risk			<0.001
No class specified	1085(68)	1055(166)	
Minor likelihood of dying	1469829(164)	1679445(310)	
Moderate likelihood of dying	371670(106)	443305(198)	
Major likelihood of dying	184975(78)	229860(125)	
Extreme likelihood of dying	72105(86)	106715(140)	

**Figure 4:**
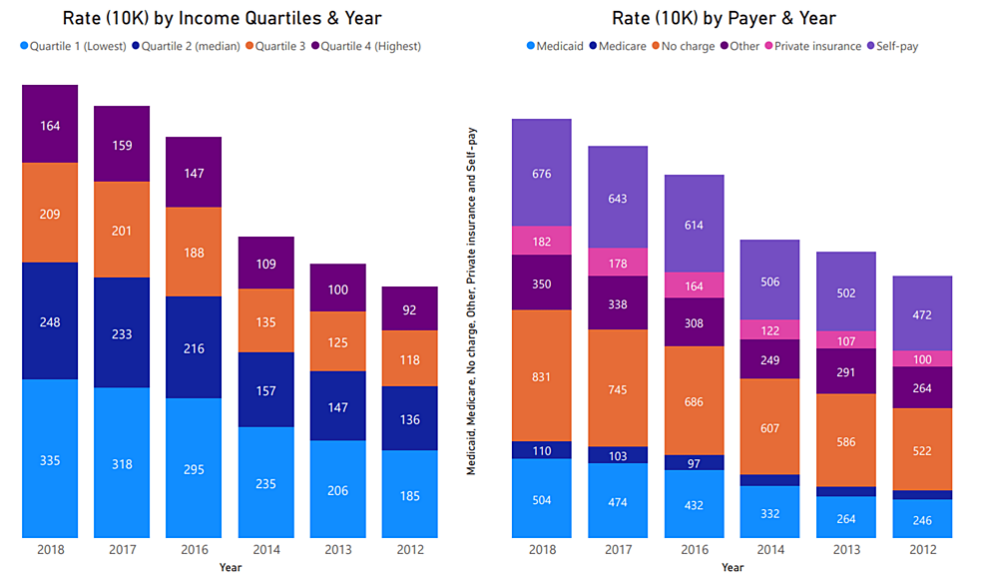
Cannabis Presentation Rate by Median Household Income (Quartiles) and Payer The rate per 10,000 presentations was consistently higher among the lowest household (lowest quartiles) income groups compared to the highest quartiles. Rate by payer consistently shows higher rates among no-charge (uninsured) followed by self-pay and Medicaid populations.

Figure 4 shows that the highest rate presentation was observed among patients without insurance coverage, indicated by "no charge," followed by self-payment and Medicaid. Presentation increased in all communities with a slightly higher rate in areas with a high population. The lowest percentile by income zip code showed higher rates of severe loss of function at presentation than other groups.

**Figure 5:**
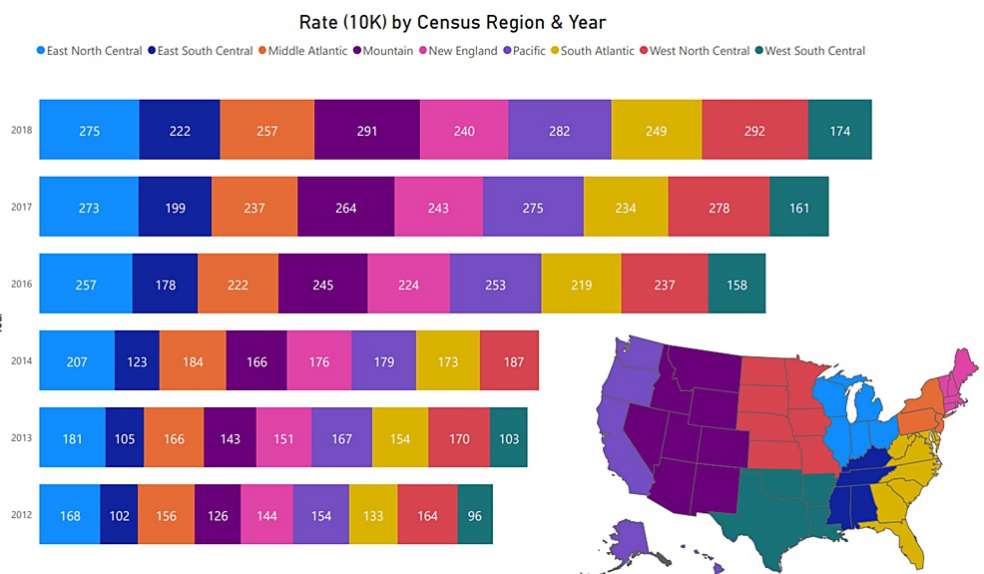
Cannabis Presentation Rate by Census Divisions of the United States. (New England): Maine, New Hampshire, Vermont, Massachusetts, Rhode Island, Connecticut (Mid-Atlantic): New York, Pennsylvania, New Jersey (East North Central): Wisconsin, Michigan, Illinois, Indiana, Ohio (West North Central): Missouri, North Dakota, South Dakota, Nebraska, Kansas, Minnesota, Iowa (South Atlantic): Delaware, Maryland, District of Columbia, Virginia, West Virginia, North Carolina, South Carolina, Georgia, Florida (East South Central) Kentucky, Tennessee, Mississippi, Alabama (West South Central) Oklahoma, Texas, Arkansas, Louisiana (Mountain) Idaho, Montana, Wyoming, Nevada, Utah, Colorado, Arizona, New Mexico (Pacific) Alaska, Washington, Oregon, California, Hawaii

The presentation rate increased across all regions, with the highest observations in East North Central, West North Central, and Mountain & Pacific Regions. The presentations among the populations tend to be on weekends (170 and 267) and weekdays (148 and 233) from 2012 to 2015 and 2016 to 2018, respectively. As shown in Figure 5, there was a consistent increase in cannabis-related hospitalization across all regions of the United States. The highest observed increases in hospitalization were in the West North Central, Mountain, Pacific, East North Central, and Middle Atlantic, respectively.

## Discussion

The consistent increase in hospitalization rate among all age demographics for cannabis-related conditions highlights the need for careful assessment to identify and mitigate the causes of such presentations at the community level. Moreover, given the established relationship with cannabis use, increased accidents, and poor psychosocial outcomes in adulthood [23], the development of public health strategies is in tandem with local legislative and legalization processes. It is imperative for concerted public health strategies. Our findings are consistent with the findings of other studies that show an increase in hospitalizations related to cannabis use [7,24]. Therefore, healthcare providers must understand the dynamics associated with a presentation to better prepare for a potential increase in cannabis-related presentations as more states legalize marijuana across the country. Understanding the presentation could serve as a vital tool for targeted educational and policy implications. It could also develop practical public health approaches for safe use, especially among youth. The overall increase in all country regions supports the argument that the trend is likely to continue. Efforts aimed at public health education and safe use in areas where it is legal and not are essential to address inpatient presentations.

As shown in this study, the rate of cannabis use among individuals with mental and alcohol use disorders continues to increase. Studies have linked cannabis use to depression, anxiety, psychosis, and other substance use [25-27]. This finding also highlights the need for further research in understanding the biological factors that directly or indirectly impact this presentation. Several studies have found the potency of cannabis used could also be related to the development of psychosis in comparison to those with no history of cannabis use [28-30]. Furthermore, research regarding the specified types of cannabis available in the legalized environments could aid in better understanding and development of regulations regarding access to more potent cannabis to the general public. Some studies have associated cannabis use and other substance use and neurologic changes in certain areas of the brain [31,32]. These studies further substantiate the need for strategic public health education programs aimed at improving the awareness and risk associated with cannabis use, especially among the younger population.

Providers, especially in the ER, urgent care centers, and primary provider settings, need to carefully access presentations that may be cannabis related and treat them accordingly. In recent years, ER presentations and hospitalization rates have increased due to edible cannabis product consumption [33-35]. The increasing trend of cannabis-associated presentations with vomiting and other psychiatric presentations presenting as comorbidities needs to be carefully and routinely accessed by providers [35,36]. Accidental consumption of cannabis through “edibles” by young children needs to be addressed through effective educational approaches and careful vigilance by adults [37]. It is also imperative for clear guidelines aimed at screening for such occurrences to better equip all providers effectively address and manage such presentations. This risk of occurrence and psychological comorbidities [38,39] need to be carefully accessed to ensure individuals at risk are targeted with coordinated public health educational initiatives at local, state, and national levels.

The findings of this study are supported by other studies that show a higher prevalence among males, minorities, and lower-income [40,41]. The presentation rate among low-income households shows the considerable financial constraints associated with smoking habits. It further highlights the need for concerted efforts aimed at understanding the drivers for such use and mitigation strategies.

## Conclusions

Cannabis use is on the rise across all regions of the United States. Inpatient presentations are not isolated to areas with higher states with cannabis legalization, medical or recreational. The use is not limited to a specific age group, even though younger individuals tend to have the highest presentation rates. Psychiatric, alcohol disorders, HIV, and burns, the rate among these populations continues to increase over the years. The disproportionate rate of extreme loss of function at presentation necessitates effective concerted public health education efforts among minority populations across the country. Partnering with school districts to create local educational programs about safe utilization. The significant cost to charge difference shows that health systems need to be active stakeholders in addressing public health education. The continuously rising cost of care necessitates a concerted approach to ensure mitigation of presentations. It is imperative to involve public health and healthcare professionals in the discussions in legislative processes aimed at cannabis legalization. Such an approach would aid in developing public health education and mitigation strategies in tandem with legislation that legalizes cannabis.
